# Scan–rescan and inter-vendor reproducibility of neurite orientation dispersion and density imaging metrics

**DOI:** 10.1007/s00234-019-02350-6

**Published:** 2019-12-27

**Authors:** Christina Andica, Koji Kamagata, Takuya Hayashi, Akifumi Hagiwara, Wataru Uchida, Yuya Saito, Kouhei Kamiya, Shohei Fujita, Toshiaki Akashi, Akihiko Wada, Masahiro Abe, Hiroshi Kusahara, Masaaki Hori, Shigeki Aoki

**Affiliations:** 1 Department of Radiology, Juntendo University Graduate School of Medicine, Tokyo, Japan; 2 Laboratory for Brain Connectomics Imaging, RIKEN Center for Biosystems Dynamics Research, Kobe, Japan; 3 Department of Radiological Sciences, Graduate School of Human Health Sciences, Tokyo Metropolitan University, Tokyo, Japan; 4 Department of Radiology, Graduate School of Medicine, The University of Tokyo, Tokyo, Japan; 5 Canon Medical Systems Corporation, Kanagawa, Japan; 6 Department of Radiology, Toho University Omori Medical Center, Tokyo, Japan

**Keywords:** Diffusion-weighted imaging, Diffusion tensor imaging, Inter-vendor reproducibility, Neurite orientation dispersion and density imaging, Scan–rescan reproducibility

## Abstract

**Purpose:**

The reproducibility of neurite orientation dispersion and density imaging (NODDI) metrics in the human brain has not been explored across different magnetic resonance (MR) scanners from different vendors. This study aimed to evaluate the scan–rescan and inter-vendor reproducibility of NODDI metrics in white and gray matter of healthy subjects using two 3-T MR scanners from two vendors.

**Methods:**

Ten healthy subjects (7 males; mean age 30 ± 7 years, range 23–37 years) were included in the study. Whole-brain diffusion-weighted imaging was performed with b-values of 1000 and 2000 s/mm^2^ using two 3-T MR scanners from two different vendors. Automatic extraction of the region of interest was performed to obtain NODDI metrics for whole and localized areas of white and gray matter. The coefficient of variation (CoV) and intraclass correlation coefficient (ICC) were calculated to assess the scan–rescan and inter-vendor reproducibilities of NODDI metrics.

**Results:**

The scan–rescan and inter-vendor reproducibility of NODDI metrics (intracellular volume fraction and orientation dispersion index) were comparable with those of diffusion tensor imaging (DTI) metrics. However, the inter-vendor reproducibilities of NODDI (CoV = 2.3–14%) were lower than the scan–rescan reproducibility (CoV: scanner A = 0.8–3.8%; scanner B = 0.8–2.6%). Compared with the finding of DTI metrics, the reproducibility of NODDI metrics was lower in white matter and higher in gray matter.

**Conclusion:**

The lower inter-vendor reproducibility of NODDI in some brain regions indicates that data acquired from different MRI scanners should be carefully interpreted.

## Introduction

Diffusion-weighted imaging (DWI) is a form of magnetic resonance (MR) imaging widely used to non-invasively evaluate the brain by measuring the displacement of water molecules in biological tissues in vivo [1, 2]. Among the available DWI techniques, diffusion tensor imaging (DTI) [3] is most commonly used to observe brain microstructural changes in neurological abnormalities [4, 5]. Although commonly used in neurological research, the use of DTI is limited in multiple ways. First, DTI is insufficient for modeling non-Gaussian diffusion scatter patterns in biological structures [6, 7]. Second, despite its sensitivity, DTI metrics are not tissue-specific. For example, a decrease in fractional anisotropy (FA) may be attributed to either or both of these: (A) loss of structural integrity (such as axonal loss or demyelination) and (B) increase in the complexity of tissue structure (such as increase in axon size and packing density and change in the degree of axonal dispersion). Finally, DTI is not the preferred method for evaluation of gray matter (GM) (particularly the cortex) because it cannot thoroughly describe microstructural abnormalities in GM due to isotropic water diffusion [8].

Neurite orientation dispersion and density imaging (NODDI) was introduced by Zhang et al. [9] to overcome the limitations of DTI. NODDI is a multi-compartment diffusion imaging model that measures microstructural metrics from multi-shell diffusion MRI data acquired using a clinical scanner within a clinically feasible period [9]. NODDI assumes a three-compartment biophysical tissue model, including intracellular (restricted diffusion; modeled by sticks), extracellular (hindered diffusion; modeled by parallel and perpendicular diffusion in an anisotropic tensor), and cerebrospinal fluid compartments (free diffusion; modeled by an isotropic tensor) within a single voxel based on the orientation-dispersed cylinder model in accordance with Watson distribution [9]. Intracellular volume fraction (ICVF) and orientation dispersion index (ODI) are the two main output metrics for NODDI that reflect neurite density and neurite orientation and dispersion, respectively, thereby disentangling the two facets of FA [9].

NODDI metrics have been identified as useful diagnostic biomarkers for revealing microstructural changes in the brains of patients with Alzheimer’s disease [10], Parkinson’s disease [7, 11, 12], stroke [13], and multiple sclerosis [14, 15]. Recently, NODDI has been used to differentiate brain tumors [16] and explore white matter (WM) microstructure in very preterm-born children [17]. In addition, NODDI has been used to demonstrate neurite properties in the human cerebral cortex that are correlated with the myeloarchitecture [18]. ICVF has exhibited good correlations with the histological measurements of hyperphosphorylated tau levels in the GM of a human tauopathy mouse model, unlike the traditional DTI metrics of mean diffusivity (MD) and FA, which failed to exhibit any correlation [19].

Chung et al. [6], Huber et al. [20], and McCunn et al. [21] attempted to assess the scan–rescan reproducibility of NODDI metrics in 8 human subjects using 1.5-T and 3-T MR scanners, a group of children (ages 7–12 years) using a 3-T MR scanner, and 10 adult Sprague–Dawley rats using a 9.4-T MR scanner, respectively; they demonstrated that ICVF and ODI are highly reproducible. However, to the best of our knowledge, no study has explored the reproducibility of NODDI metrics in the human brain across different MR scanners from different vendors to date. Therefore, in this study, we aimed to evaluate the scan–rescan and inter-vendor reproducibility of NODDI metrics in WM and GM of healthy subjects using two 3-T MR scanners from two different vendors.

## Materials and methods

### Study participants

A total of 10 healthy subjects (7 males and 3 females; mean age 30 ± 7 years, range 23–37 years) with no history of neurological, psychiatric, or other systemic diseases were included in the study. The Institutional Review Board of Juntendo University Hospital, Tokyo, Japan approved this study, and all subjects gave written informed consent prior to participation.

### Imaging protocol

Each subject was scanned on two sessions scheduled at least 1 day apart using two 3-T MRI scanners (Vantage Galan ZGO, Canon Medical Systems, Otawara, Japan (scanner A) and MAGNETOM Prisma, Siemens Healthcare, Erlangen, Germany (scanner B)), both located at one site. All subjects were scanned twice in each session to assess the scan–rescan reproducibility. Each volunteer was removed from the scanner briefly following the first acquisition and repositioned for the second acquisition.

Whole-brain DWI was acquired using a 2D multiband spin-echo echo-planar imaging (EPI) sequence [22] with b-values of 1000 and 2000 s/mm^2^, each with 64 motion-probing directions. Each DWI acquisition was completed with one b0 image without diffusion gradients. Standard and reverse phase-encoded blipped images without diffusion weighting (blip up and blip down) were also acquired to correct for magnetic susceptibility-induced distortions related to EPI acquisitions [23]. We also obtained 3D T1-weighted image with magnetization-prepared rapid gradient echo (MPRAGE) with 180° radiofrequency pulse. The sequence parameters of each scanner are shown in Table 1.

**Table 1:** Acquisition parameters

	DWI		T1 MPRAGE	
	Scanner A	Scanner B	Scanner A	Scanner B
TR/TE (ms)	4900/70	4900/70	2400/2.7	2300/2.32
FOV (mm)	230 × 230	230 × 230	240 × 240	240 × 240
Matrix size	128 × 128	128 × 128	256 × 256	256 × 256
Resolution (mm)	1.8 × 1.8	1.8 × 1.8	0.9 × 0.9	0.9 × 0.9
Slice thickness (mm)	1.8	1.8	0.9	0.9
Acquisition time (min)	11.07	10.56	5.08	6.25
Gradient direction	64	64		
Maximal gradient strength (mT/m)	100	80		
Flip angle (°)	90	90		
Acceleration factor	2	2		
Multiband factor	2	2		

*FOV*, field of view; *TE*, echo time; *TR*, repetition time

### Pre-processing for diffusion MRI

The diffusion MRI data were corrected for susceptibility-induced geometric distortions, eddy current distortions, and inter-volume subject motion using EDDY and TOPUP toolboxes [23]. Next, all diffusion MRI data were visually checked for 64 different directions in axial, sagittal, and coronal planes for both scanners. We confirmed that all data were free from severe artifacts, such as gross geometric distortion, signal dropout, and bulk motion.

The resulting images were fitted to the NODDI model [9] using the NODDI MATLAB Toolbox 5 (http://www.nitrc.org/projects/noddi_toolbox); then, ICVF, ODI, and isotropic volume fraction (ISO) maps were generated. The diffusion tensor was estimated using ordinary least squares applied to diffusion-weighted images with b-values of 0 and 1000 s/mm^2^. FA, MD, axial diffusivity (AD), and radial diffusivity (RD) maps were then generated for all subjects using the DTIFIT tool implemented in functional magnetic resonance imaging of the brain (FMRIB) Software Library 5.0.9 (FSL, Oxford Centre for Functional MRI of the Brain, UK; www.fmrib.ox.ac.uk/fsl) to fit the tensor model to each voxel of the DWI data [3].

### Signal-to-noise ratio calculation

**Fig. 1:**
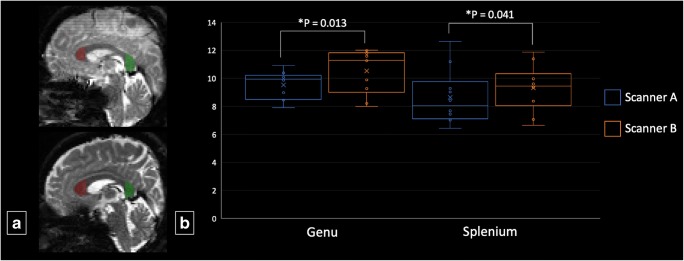
**a** Placement of regions of interests on b0 images of the genu (red) and splenium (green) of the corpus callosum for the measurement of signal-to-noise ratio. **b** Boxplots of group mean signal-to-noise ratio for scanners A and B in the genu and splenium. *Wilcoxon signed-rank test significance at *P* < 0.05

Signal-to-noise ratio (SNR) was calculated for each scanner using the single region of interest (ROI) approach and two b0 images with Camino [24]. Manual ROIs were drawn in the genu and splenium of the corpus callosum on the b0 images in the sagittal plane obtained on the first scan for each scanner (Fig. 1a). First, σ_diff_ was calculated as follows:

$$ {\sigma}_{\mathrm{diff}}=\frac{stddev\ \left({S}_{\left\{i1\right\}}-{S}_{\left\{i2\right\}},\dots, {S}_{\left\{N1\right\}}-{S}_{\left\{N2\right\}}\right)}{sqrt(2)} $$

where S{i1} is the signal from voxel i of image 1, whereas S{i2} is the signal from the same voxel in image 2, and N represents the number of voxels in an ROI. Then, SNR was calculated as the mean signal from the ROI divided by σ_diff_, as follows:

$$ {SNR}_{\mathrm{diff}}=\frac{mean\ \left({S}_{\left\{i1\right\}}+{S}_{\left\{i2\right\}}\right)}{2.0\times {\sigma}_{\mathrm{diff}}} $$

### ROI analysis

DTI and NODDI values were measured for the whole WM and GM, subcortical GM, localized WM, and cortical regions. Whole WM and GM, subcortical GM, and cortical segmentation were performed with FreeSurfer pipeline (http://surfer.nmr.mgh.harvard.edu/fswiki) as previously described [25] using 3D MPRAGE T1-WI.

Whole WM and GM, subcortical GM (caudate, putamen, pallidum, thalamus, hippocampus, amygdala, and accumbent), and cortical (frontal, temporal, parietal, occipital, and cingulate) regions were then labeled using the Desikan–Killiany atlas [26]. For localized WM areas, FA maps of all subjects were first realigned according to the FA template of the Johns Hopkins University International Consortium for Brain Mapping (JHU ICBM) using the FMRIB’s nonlinear image registration tool [27]. The corresponding MD, AD, RD, ICVF, ODI, and ISO maps were subsequently realigned according to the transformation parameter obtained from the FA maps. Localized WM (genu, body, and splenium of the corpus callosum, corticospinal tract, anterior and posterior limb of internal capsule, anterior, superior, and posterior corona radiata; posterior thalamic radiation; sagittal stratum; external capsule; superior longitudinal fasciculus, superior fronto-occipital fasciculus; and uncinate fasciculus) regions were labeled with JHU ICBM-DTI-81 WM labels [28]. Lastly, the average diffusion metric was averaged over the region delineated by those atlases for all subjects.

### Statistical analysis

All statistical analyses were performed using the IBM SPSS Statistics for Windows (version 22.0; IBM Corporation, Armonk, NY, USA). The Shapiro–Wilk test was used to assess the normality of the SNR data. Not all data were normally distributed; therefore, differences in SNR measured in the genu and splenium of the corpus callosum between scanners A and B from the first scan were analyzed using the Wilcoxon signed-rank test. The threshold for statistical significance was set at *p* value < 0.05.

Coefficient of variation (CoV) was determined to evaluate the scan–rescan and inter-vendor reproducibility using the following equation:

$$ CoV\ \left(\%\right)=\left( Standard\ deviation/ Mean\right)\times 100 $$

The inter-vendor CoV was calculated using the average values from each scanner. For each subject, the inter-vendor CoVs were calculated using the data of the first scan, which was averaged into a single inter-scanner CoV value. The scan–rescan CoVs were calculated for each subject and then averaged across all subjects.

In addition, we used intraclass correlation coefficient (ICC) with 95% confidence interval. ICC values less than 0.50 were indicative of poor reliability, values between 0.50 and 0.75 were indicative of moderate reliability, values between 0.75 were indicative of good reliability, and values greater than 0.90 were indicative of excellent reliability [29].

## Results

**Fig. 2:**
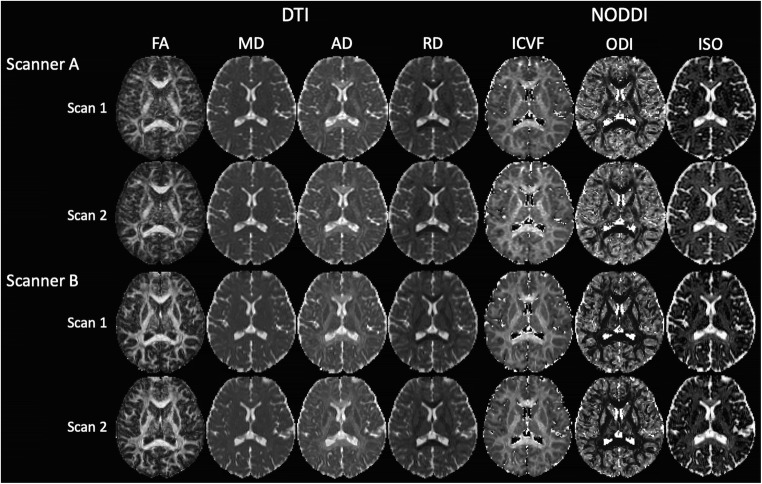
Diffusion tensor imaging (fractional anisotropy [FA], mean diffusivity [MD], axial diffusivity [AD], and radial diffusivity [RD]) and neurite orientation dispersion and density imaging (intracellular volume fraction [ICVF], orientation dispersion index [ODI], and isotropic volume fraction [ISO]) maps of one healthy subject

**Fig. 3:**
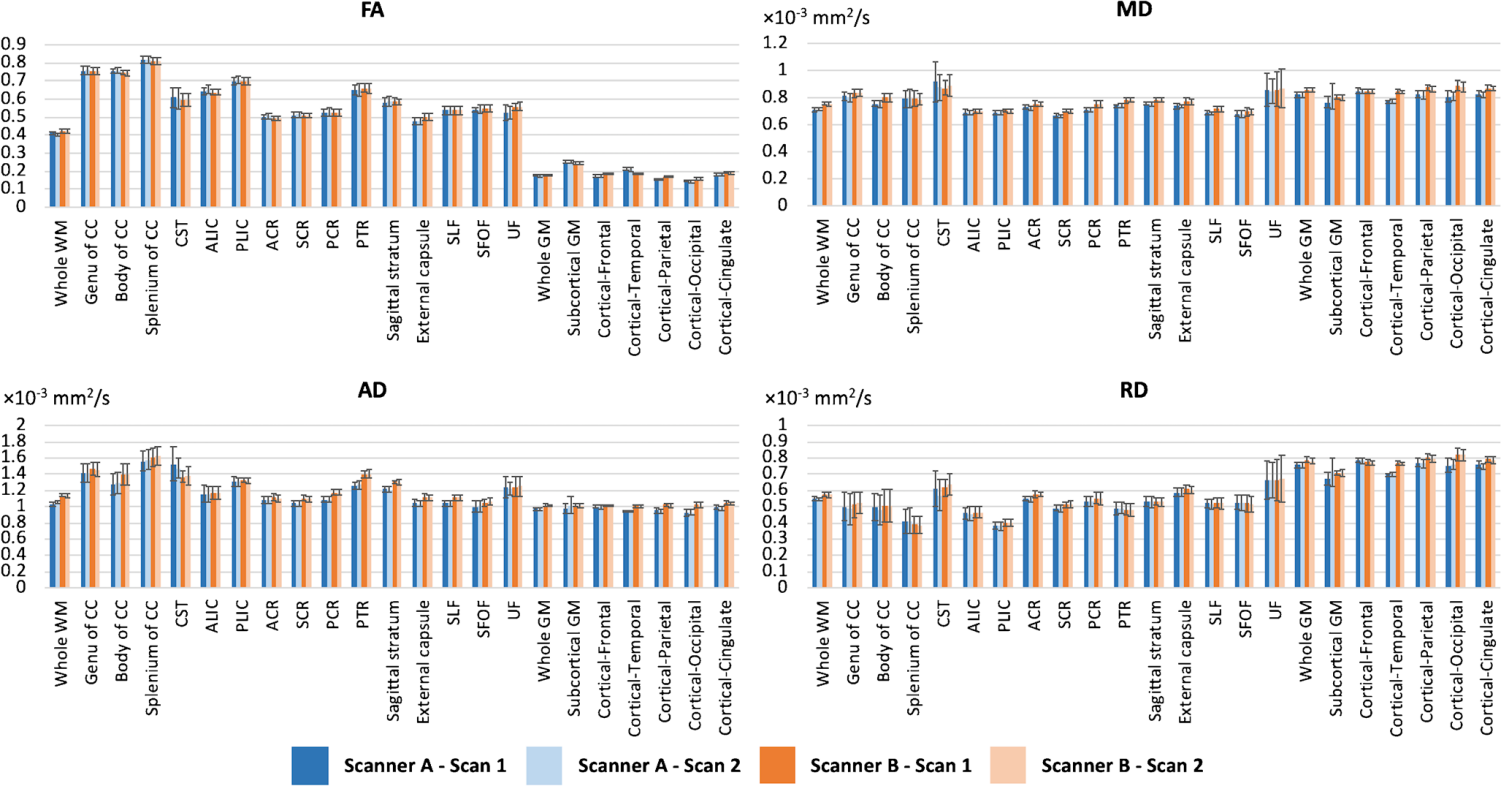
Means and standard deviations of diffusion tensor imaging (fractional anisotropy [FA], mean diffusivity [MD], axial diffusivity [AD], and radial diffusivity [RD]) metrics across all subjects. Abbreviations: ACR anterior corona radiata, ALIC anterior limb of internal capsule, CC corpus callosum, CST corticospinal tract, GM gray matter, PCR posterior corona radiata, PLIC posterior limb of internal capsule, PTR posterior thalamic radiation, SCR superior corona radiata, SFOF superior fronto-occipital fasciculus, SLF superior longitudinal fasciculus, UF uncinate fasciculus, WM white matter

**Fig. 4:**
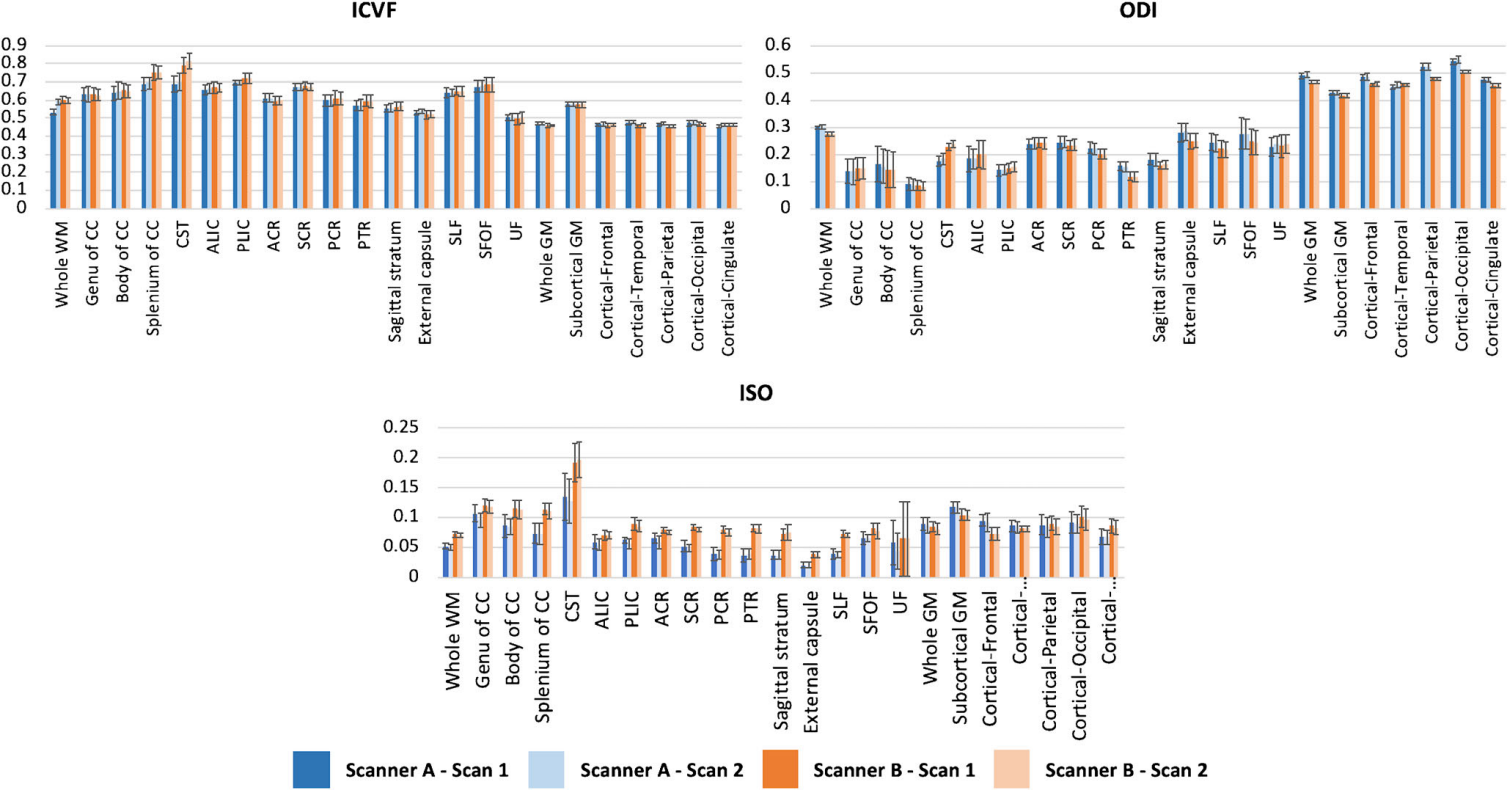
Means and standard deviations of neurite orientation dispersion and density imaging (intracellular volume fraction (ICVF), orientation dispersion index (ODI), and isotropic volume fraction [ISO]) metrics across all subjects. Abbreviations: ACR anterior corona radiata, ALIC anterior limb of internal capsule, CC corpus callosum, CST corticospinal tract, GM gray matter, PCR posterior corona radiata, PLIC posterior limb of internal capsule, PTR posterior thalamic radiation, SCR superior corona radiata, SFOF superior fronto-occipital fasciculus, SLF superior longitudinal fasciculus, UF uncinate fasciculus, WM white matter

For the genu and splenium, the SNR of scanner A was significantly lower than that of scanner B (Fig. 1b). The scan–rescan DTI and NODDI maps of one healthy participant for the two scanners are represented in Fig. 2. Figures 3 and 4 present the mean values of DTI and NODDI metrics, respectively, for WM and GM.

Table 2 shows the scan–rescan CoVs of DTI and NODDI metrics. In WM, the highest scan–rescan CoVs were 1.9% (FA), 3.3% (MD), 2.5% (AD), and 4.1% (RD) and 1.4% (ICVF), 3.8% (ODI), and 18.5% (ISO) for DTI and NODDI metrics, respectively, in scanner A. On the other hand, in scanner B, the CoVs were 0.9% (FA), 1.7% (MD), 1.3% (AD), and 2.0% (RD) and 1.5% (ICVF), 2.6% (ODI), and 9.1% (ISO) for DTI and NODDI metrics, respectively. In GM, the highest scan–rescan CoVs were 2.0% (FA), 2.7% (MD), 2.1% (AD), and 3.1% (RD), and 0.8% (ICVF), 0.8% (ODI), and 4.8% (ISO) for DTI and NODDI metrics, respectively, in scanner A. On the other hand, in scanner B, the CoVs were 0.5% (FA), 0.8% (MD), 0.8% (AD), and 0.8% (RD) and 0.8% (ICVF), 0.5% (ODI), and 3.5% (ISO) for DTI and NODDI metrics, respectively.

**Table 2:** Scan–rescan coefficient of variation (CoV [%]) across all subjects

	Scanner A							Scanner B						
	DTI				NODDI			DTI				NODDI		
	FA	MD	AD	RD	ICVF	ODI	ISO	FA	MD	AD	RD	ICVF	ODI	ISO
White matter														
Whole WM	0.5 ± 0.5	0.2 ± 0.2	0.2 ± 0.2	0.4 ± 0.3	0.4 ± 0.3	0.3 ± 0.2	3.6 ± 2.7	0.5 ± 0.5	0.2 ± 0.1	0.1 ± 0.1	0.2 ± 0.1	0.4 ± 0.2	0.2 ± 0.2	1.6 ± 1.1
Genu of CC	0.3 ± 0.3	0.9 ± 0.5	1.0 ± 0.7	2.5 ± 2.1	0.9 ± 0.7	2.6 ± 1.7	5.8 ± 4.7	0.3 ± 0.2	0.5 ± 0.3	0.6 ± 0.4	1.5 ± 1.2	0.9 ± 0.5	1.4 ± 1.0	3.3 ± 2.7
Body of CC	0.4 ± 0.3	0.6 ± 0.3	1.1 ± 1.2	2.6 ± 2.2	0.8 ± 0.7	3.8 ± 4.3	4.3 ± 3.1	0.3 ± 0.1	0.4 ± 0.3	0.5 ± 0.4	0.9 ± 0.8	0.8 ± 0.8	1.9 ± 1.5	2.6 ± 3.1
Splenium of CC	0.3 ± 0.3	1.1 ± 0.7	0.9 ± 0.6	2.6 ± 1.9	0.6 ± 0.3	2.3 ± 1.8	5.3 ± 3.8	0.2 ± 0.2	1.1 ± 1.2	0.8 ± 0.6	2.0 ± 2.1	0.5 ± 0.4	1.5 ± 1.0	3.8 ± 3.8
CST	1.2 ± 0.6	3.3 ± 3.8	2.5 ± 3.0	4.1 ± 4.1	1.4 ± 1.2	2.8 ± 1.5	5.3 ± 3.2	0.7 ± 0.6	1.7 ± 1.8	1.3 ± 1.5	2.0 ± 1.4	1.5 ± 0.9	2.0 ± 1.8	2.9 ± 3.2
ALIC	0.6 ± 0.7	0.8 ± 0.5	1.0 ± 0.9	1.7 ± 1.2	0.7 ± 0.8	2.4 ± 1.8	7.1 ± 4.7	0.3 ± 0.2	0.7 ± 0.4	0.7 ± 0.4	1.1 ± 0.7	0.9 ± 0.4	1.2 ± 1.1	5.4 ± 3.7
PLIC	0.6 ± 0.3	0.6 ± 0.5	0.5 ± 0.4	1.2 ± 0.8	0.7 ± 0.4	0.7 ± 0.6	6.8 ± 5.7	0.3 ± 0.2	0.6 ± 0.6	0.7 ± 0.6	0.8 ± 0.6	0.6 ± 0.5	1.6 ± 1.1	2.4 ± 1.1
ACR	0.7 ± 0.6	0.6 ± 0.4	0.7 ± 0.4	0.9 ± 0.8	0.6 ± 0.5	0.8 ± 0.5	7.7 ± 6.6	0.2 ± 0.1	0.3 ± 0.3	0.5 ± 0.4	0.5 ± 0.5	0.7 ± 0.6	0.8 ± 0.6	2.9 ± 3.2
SCR	0.8 ± 0.3	0.7 ± 0.3	0.4 ± 0.3	0.9 ± 0.5	0.5 ± 0.3	0.7 ± 0.5	6.8 ± 4.2	0.4 ± 0.2	0.4 ± 0.3	0.3 ± 0.3	0.4 ± 0.4	0.8 ± 0.4	0.4 ± 0.2	2.6 ± 1.7
PCR	0.4 ± 0.3	0.4 ± 0.3	0.3 ± 0.3	1.2 ± 0.5	0.5 ± 0.3	1.2 ± 0.9	6.4 ± 6.0	0.3 ± 0.2	0.3 ± 0.3	0.3 ± 0.2	0.7 ± 0.3	0.7 ± 0.4	0.8 ± 0.6	3.5 ± 1.9
PTR	0.3 ± 0.3	0.5 ± 0.1	0.6 ± 0.3	1.1 ± 0.8	0.4 ± 0.3	1.0 ± 0.9	8.7 ± 4.7	0.2 ± 0.1	0.3 ± 0.2	0.5 ± 0.4	0.7 ± 0.3	0.5 ± 0.4	1.5 ± 1.2	2.1 ± 2.1
Sagittal stratum	0.4 ± 0.2	0.5 ± 0.4	0.2 ± 0.1	0.9 ± 1.1	0.6 ± 0.4	1.7 ± 1.4	9.1 ± 6.0	0.5 ± 0.3	0.4 ± 0.3	0.5 ± 0.4	0.6 ± 0.3	0.7 ± 0.3	1.0 ± 0.7	5.8 ± 4.3
External capsule	0.6 ± 0.5	0.6 ± 0.5	0.6 ± 0.4	1.0 ± 0.9	0.8 ± 0.5	1.3 ± 1.1	7.0 ± 5.6	0.3 ± 0.2	0.4 ± 0.2	0.4 ± 0.2	0.6 ± 0.5	0.8 ± 0.5	0.7 ± 0.8	3.5 ± 3.5
SLF	0.5 ± 0.5	0.4 ± 0.3	0.3 ± 0.2	0.9 ± 0.7	0.4 ± 0.4	1.3 ± 0.7	5.4 ± 3.8	0.3 ± 0.2	0.3 ± 0.3	0.3 ± 0.2	0.5 ± 0.4	0.5 ± 0.4	0.7 ± 0.4	3.9 ± 1.7
SFOF	1.3 ± 0.7	0.6 ± 0.5	0.5 ± 0.5	1.3 ± 0.9	0.6 ± 0.4	1.8 ± 1.7	5.5 ± 3.0	0.9 ± 0.7	0.9 ± 0.6	1.1 ± 0.6	1.2 ± 1.0	1.2 ± 0.7	1.2 ± 0.9	9.1 ± 4.3
UF	1.9 ± 1.5	1.8 ± 1.5	1.6 ± 1.4	2.4 ± 1.8	1.3 ± 0.8	3.3 ± 2.7	18.5 ± 16.7	0.7 ± 0.4	1.4 ± 1.1	1.0 ± 0.6	1.7 ± 1.4	1.1 ± 0.7	2.6 ± 1.7	8.7 ± 6.5
Gray matter														
Whole GM	1.2 ± 1.0	0.3 ± 0.2	0.3 ± 0.2	0.3 ± 0.2	0.5 ± 0.5	0.5 ± 0.5	2.9 ± 3.0	0.3 ± 0.2	0.4 ± 0.2	0.3 ± 0.2	0.4 ± 0.2	0.4 ± 0.4	0.2 ± 0.1	1.9 ± 1.4
Sub-cortical	1.2 ± 1.3	2.7 ± 3.1	2.1 ± 2.7	3.1 ± 3.5	0.7 ± 0.6	0.4 ± 0.2	1.7 ± 1.2	0.4 ± 0.2	0.8 ± 0.9	0.8 ± 1.0	0.8 ± 0.9	0.4 ± 0.3	0.2 ± 0.1	1.0 ± 0.9
Cortical frontal	1.3 ± 1.6	0.4 ± 0.3	0.4 ± 0.3	0.5 ± 0.4	0.8 ± 0.6	0.5 ± 0.7	4.2 ± 4.5	0.3 ± 0.2	0.4 ± 0.3	0.3 ± 0.2	0.4 ± 0.3	0.6 ± 0.3	0.2 ± 0.1	2.2 ± 1.5
Cortical temporal	1.9 ± 0.8	0.3 ± 0.3	0.2 ± 0.2	0.5 ± 0.3	0.7 ± 0.8	0.8 ± 0.6	4.8 ± 4.0	0.5 ± 0.3	0.3 ± 0.1	0.3 ± 0.1	0.4 ± 0.1	0.5 ± 0.4	0.2 ± 0.1	1.3 ± 0.8
Cortical parietal	0.8 ± 0.8	0.5 ± 0.3	0.5 ± 0.3	0.5 ± 0.3	0.4 ± 0.3	0.3 ± 0.4	2.7 ± 1.9	0.4 ± 0.4	0.6 ± 0.3	0.6 ± 0.2	0.7 ± 0.3	0.5 ± 0.4	0.2 ± 0.2	3.5 ± 2.3
Cortical occipital	2.0 ± 0.9	0.6 ± 0.3	0.4 ± 0.3	0.7 ± 0.3	0.5 ± 0.4	0.6 ± 0.4	2.2 ± 1.7	0.4 ± 0.4	0.4 ± 0.3	0.4 ± 0.2	0.5 ± 0.3	0.8 ± 0.5	0.5 ± 0.3	3.2 ± 1.2
Cortical cingulate	1.5 ± 0.8	0.8 ± 0.4	0.5 ± 0.4	0.9 ± 0.4	0.3 ± 0.3	0.3 ± 0.3	2.4 ± 1.7	0.5 ± 0.3	0.4 ± 0.2	0.4 ± 0.3	0.4 ± 0.2	0.3 ± 0.3	0.3 ± 0.3	2.2 ± 1.5

*ACR*, anterior corona radiata; *ALIC*, anterior limb of the internal capsule; *CC*, corpus callosum; *CST*, corticospinal tract; *GM*, gray matter; *PCR*, posterior corona radiata; *PLIC*, posterior limb of the internal capsule; *PTR*, posterior thalamic radiation; *SCR*, superior corona radiata; *SFOF*, superior fronto-occipital fasciculus; *SLF*, superior longitudinal fasciculus; *UF*, uncinate fasciculus; *WM*, white matter

Table 3 shows the inter-vendor CoVs of DTI and NODDI metrics. For all metrics, the inter-vendor CoVs were generally higher than the scan–rescan CoVs. In WM, the highest inter-vendor CoVs for DTI metrics were 2.6% (FA), 3.9% (MD), 5.3% (AD), and 5.9% (RD), whereas those for NODDI metrics were 7.0% (ICVF), 14% (ODI), and 38.9% (ISO). In GM, the highest inter-vendor CoVs for DTI metrics were 7.1% (FA), 4.7% (MD), 5.1% (AD), and 5.0% (RD), whereas those for NODDI metrics were 2.3% (ICVF), 4.3% (ODI), and 13.9% (ISO).

**Table 3:** Inter-vendor coefficient of variation (CoV [%]) across all subjects

	DTI			NODDI			
	FA	MD	AD	RD	ICVF	ODI	ISO
White matter							
Whole WM	2.1 ± 0.7	3.0 ± 0.4	3.8 ± 0.3	2.2 ± 0.6	0.9 ± 0.4	4.2 ± 0.6	15.9 ± 4.4
Genu of CC	0.5 ± 0.4	1.4 ± 0.5	2.3 ± 1.3	2.5 ± 2.4	1.5 ± 1.2	5.2 ± 4.4	7.5 ± 4.4
Body of CC	0.6 ± 0.4	2.7 ± 1.1	4.8 ± 1.3	3.2 ± 2.8	1.3 ± 1.0	7.3 ± 6.9	16.2 ± 7.5
Splenium of CC	0.4 ± 0.4	1.4 ± 1.2	2.6 ± 1.5	5.9 ± 3.3	4.6 ± 1.1	5.2 ± 4.1	21.6 ± 7.2
CST	2.3 ± 1.2	3.9 ± 3.7	5.3 ± 4.0	4.0 ± 3.3	7.0 ± 2.6	10.2 ± 3.3	21.1 ± 10.0
ALIC	0.8 ± 0.6	0.8 ± 0.4	1.6 ± 1.2	1.8 ± 1.5	1.4 ± 1.1	4.7 ± 2.9	12.2 ± 7.1
PLIC	0.5 ± 0.3	1.3 0.7	1.2 ± 0.7	2.0 ± 0.6	2.3 ± 0.9	2.9 ± 1.3	18.5 ± 6.6
ACR	0.8 ± 0.6	1.7 ± 0.5	1.5 ± 0.9	2.1 ± 1.2	1.3 ± 1.2	1.6 ± 1.1	10.7 ± 6.5
SCR	0.6 ± 0.6	2.4 ± 0.6	2.9 ± 0.5	1.9 ± 0.9	1.5 ± 0.6	2.2 ± 0.8	23.4 ± 9.9
PCR	0.8 ± 0.5	2.8 ± 0.7	4.1 ± 0.6	1.6 ± 0.9	1.5 ± 0.6	5.9 ± 2.0	34.9 ± 11.4
PTR	1.2 ± 0.8	2.6 ± 1.0	5.3 ± 1.4	1.8 ± 1.3	2.5 ± 1.6	14.0 ± 4.2	38.9 ± 10.4
Sagittal stratum	1.2 ± 1.0	1.9 ± 0.5	3.9 ± 1.2	1.7 ± 0.9	1.8 ± 0.9	6.8 ± 2.9	31.9 ± 11.5
External capsule	2.6 ± 0.9	2.5 ± 0.7	3.3 ± 0.8	2.1 ± 0.9	1.8 ± 1.1	5.3 ± 2.4	29.1 ± 8.1
SLF	0.7 ± 0.7	1.9 ± 0.6	3.1 ± 0.7	1.4 ± 0.7	1.3 ± 0.6	5.0 ± 2.3	30.4 ± 9.8
SFOF	2.1 ± 1.0	1.4 ± 0.7	2.9 ± 1.5	2.4 ± 1.7	2.0 ± 1.6	6.6 ± 2.1	11.0 ± 6.0
UF	2.5 ± 1.9	3.5 ± 2.9	2.7 ± 2.7	4.2 ± 3.4	3.4 ± 2.8	4.1 ± 3.3	28.7 ± 19.7
Gray matter							
Whole GM	1.4 ± 1.0	2.2 ± 0.5	2.5 ± 0.5	2.0 ± 0.6	1.1 ± 0.4	2.4 ± 0.7	5.2 ± 2.1
Sub-cortical	1.9 ± 1.3	3.1 ± 1.5	2.9 ± 1.1	3.6 ± 1.3	0.8 ± 0.7	1.2 ± 0.8	6.2 ± 3.1
Cortical frontal	4.1 ± 2.0	0.9 ± 0.6	0.8 ± 0.7	1.2 ± 0.8	0.7 ± 0.3	2.8 ± 1.2	13.9 ± 6.7
Cortical temporal	7.1 ± 1.8	4.4 ± 0.4	3.3 ± 0.4	5.0 ± 0.4	2.3 ± 0.8	0.8 ± 0.6	3.8 ± 3.8
Cortical parietal	5.0 ± 1.3	2.7 ± 0.8	3.5 ± 0.8	2.1 ± 0.8	1.3 ± 0.8	4.3 ± 0.7	3.9 ± 3.4
Cortical occipital	3.1 ± 1.4	4.7 ± 0.7	5.1 ± 0.8	4.4 ± 0.7	1.2 ± 0.7	3.6 ± 0.9	6.6 ± 3.5
Cortical cingulate	3.1 ± 1.5	2.4 ± 0.7	2.8 ± 0.6	2.1 ± 0.7	0.9 ± 0.6	2.1 ± 0.6	12.3 ± 5.5

*ACR*, anterior corona radiata; *ALIC*, anterior limb of the internal capsule; *CC*, corpus callosum; *CST*, corticospinal tract; *GM*, gray matter; *PCR*, posterior corona radiata; *PLIC*, posterior limb of the internal capsule; *PTR*, posterior thalamic radiation; *SCR*, superior corona radiata; *SFOF*, superior fronto-occipital fasciculus; *SLF*, superior longitudinal fasciculus; *UF*, uncinate fasciculus; *WM*, white matter

Table 4 shows the scan–rescan ICCs of DTI and NODDI metrics. In WM, scanner A and scanner B demonstrated poor to excellent scan–rescan reproducibility of DTI (scanner A: FA [ICC = 0.744–0.995], MD [ICC = 0.777–0.968], AD [ICC = 0.964–0.994], and RD [ICC = 0.884–0.980]; scanner B: FA [ICC = 0.918–0.996], MD [ICC = 0.905–0.987], AD [ICC = 0.912–0.997], and RD [ICC = 0.926–0.997]) and NODDI metrics (scanner A: ICVF [ICC = 0.773–0.989], ODI [ICC = 0.910–0.996], and ISO [ICC = 0.211–0.945]; scanner B: ICVF [ICC = 0.909–0.987], ODI [ICC = 0.789–0.998], and ISO [ICC = 0.133–0.997]). In GM, scanner A and B also demonstrated poor to excellent scan–rescan reproducibility of DTI (scanner A: FA [ICC = 0.383–0.890], MD [ICC = 0.731–0.980], AD [ICC = 0.769–0.985], and RD [ICC = 0.690–0.974]; scanner B: FA [ICC = 0.810–0.984], MD [ICC = 0.593–0.956], AD [ICC = 0.450–0.983], and RD [ICC = 0.703–0.984]) and NODDI metrics (scanner A: ICVF [ICC = 0.668–0.952], ODI [ICC = 0.729–0.926], and ISO [ICC = 0.396–0.975]; scanner B: ICVF [ICC = 0.812–0.963], ODI [ICC = 0.929–0.976], and ISO [ICC = 0.915–0.972]).

**Table 4:** Scan–rescan intraclass correlation coefficient across all subjects

	Scanner A							Scanner B						
	DTI				NODDI			DTI			NODDI			
	FA	MD	AD	RD	ICVF	ODI	ISO	FA	MD	AD	RD	ICVF	ODI	ISO
White matter														
Whole WM	0.923	0.963	0.974	0.939	0.964	0.977	0.747	0.996	0.987	0.991	0.990	0.980	0.988	0.895
Genu of CC	0.978	0.949	0.975	0.980	0.968	0.990	0.676	0.985	0.976	0.987	0.986	0.974	0.997	0.710
Body of CC	0.901	0.948	0.983	0.974	0.973	0.989	0.922	0.967	0.987	0.997	0.997	0.959	0.998	0.942
Splenium of CC	0.971	0.968	0.986	0.975	0.985	0.985	0.924	0.984	0.865	0.980	0.951	0.982	0.993	0.765
CST	0.979	0.822	0.832	0.884	0.905	0.910	0.945	0.988	0.879	0.898	0.926	0.890	0.789	0.943
ALIC	0.913	0.777	0.969	0.959	0.922	0.984	0.819	0.977	0.837	0.990	0.959	0.943	0.997	0.133
PLIC	0.939	0.783	0.975	0.955	0.812	0.996	0.211	0.994	0.901	0.918	0.972	0.964	0.980	0.938
ACR	0.869	0.904	0.964	0.825	0.946	0.985	0.621	0.991	0.966	0.980	0.926	0.909	0.988	0.378
SCR	0.896	0.811	0.974	0.917	0.939	0.993	0.692	0.976	0.959	0.985	0.985	0.912	0.997	0.510
PCR	0.987	0.957	0.979	0.978	0.983	0.986	0.821	0.987	0.980	0.989	0.993	0.982	0.989	0.692
PTR	0.995	0.925	0.970	0.968	0.989	0.986	0.792	0.995	0.973	0.971	0.989	0.987	0.978	0.805
Sagittal stratum	0.990	0.880	0.994	0.951	0.948	0.974	0.524	0.957	0.936	0.841	0.978	0.966	0.979	0.711
External capsule	0.958	0.845	0.969	0.909	0.816	0.978	0.781	0.988	0.975	0.988	0.968	0.947	0.993	0.868
SLF	0.970	0.924	0.987	0.967	0.970	0.990	0.844	0.993	0.979	0.989	0.993	0.977	0.996	0.634
SFOF	0.744	0.956	0.987	0.978	0.984	0.993	0.557	0.918	0.905	0.912	0.968	0.960	0.995	0.149
UF	0.872	0.952	0.911	0.969	0.773	0.914	0.884	0.962	0.987	0.985	0.990	0.954	0.969	0.997
Gray matter														
Whole GM	0.739	0.978	0.978	0.968	0.790	0.877	0.911	0.972	0.941	0.937	0.942	0.892	0.961	0.950
Subcortical	0.383	0.731	0.769	0.690	0.775	0.918	0.922	0.984	0.593	0.450	0.703	0.963	0.976	0.972
Cortical frontal	0.764	0.935	0.937	0.911	0.678	0.870	0.807	0.983	0.931	0.890	0.938	0.897	0.968	0.956
Cortical temporal	0.810	0.928	0.967	0.885	0.668	0.729	0.396	0.810	0.942	0.953	0.941	0.900	0.968	0.940
Cortical parietal	0.890	0.971	0.973	0.968	0.866	0.962	0.975	0.938	0.911	0.973	0.905	0.835	0.942	0.915
Cortical occipital	0.806	0.980	0.985	0.974	0.952	0.867	0.970	0.979	0.983	0.983	0.984	0.812	0.944	0.971
Cortical cingulate	0.776	0.905	0.911	0.901	0.694	0.893	0.973	0.960	0.956	0.939	0.966	0.930	0.929	0.963

*ACR*, anterior corona radiata; *ALIC*, anterior limb of the internal capsule; *CC*, corpus callosum; *CST*, corticospinal tract; *GM*, gray matter; *PCR*, posterior corona radiata; *PLIC*, posterior limb of the internal capsule; *PTR*, posterior thalamic radiation; *SCR*, superior corona radiata; *SFOF*, superior fronto-occipital fasciculus; *SLF*, superior longitudinal fasciculus; *UF*, uncinate fasciculus; *WM*, white matter

Table 5 shows the inter-vendor ICCs of DTI and NODDI metrics. In WM, DTI and NODDI metrics showed poor to excellent inter-vendor reproducibility (DTI: FA [ICC = 0.538–0.973], MD [ICC = 0.214–0.890], AD [ICC = 0.119–0.929], and RD [ICC = 0.411–0.949]; NODDI: ICVF [ICC = 0.300–0.935], ODI [ICC = 0.181–0.962], and ISO [ICC = 0.013–0.545]). In GM, DTI metrics showed poor to moderate inter-vendor reproducibility (FA [ICC = 0.013–0.528], MD [ICC = 0.095–0.488], AD [ICC = 0.133–0.416], and RD [ICC = 0.084–0.596]) and NODDI metrics showed poor to excellent inter-vendor reproducibility (ICVF [ICC = 0.395–0.849], ODI [ICC = 0.043–0.580], and ISO [ICC = 0.092–0.903]).

**Table 5:** Inter-vendor intraclass correlation coefficient across all subjects

	DTI				NODDI		
	FA	MD	AD	RD	ICVF	ODI	ISO
White matter							
Whole WM	0.538	0.214	0.150	0.429	0.869	0.224	0.018
Genu of CC	0.938	0.860	0.846	0.941	0.859	0.949	0.545
Body of CC	0.755	0.557	0.816	0.949	0.934	0.962	0.310
Splenium of CC	0.917	0.890	0.872	0.825	0.554	0.902	0.211
CST	0.908	0.589	0.482	0.565	0.300	0.181	0.248
ALIC	0.860	0.722	0.929	0.842	0.895	0.948	0.324
PLIC	0.969	0.547	0.856	0.833	0.445	0.903	0.013
ACR	0.824	0.576	0.846	0.411	0.756	0.932	0.303
SCR	0.950	0.386	0.614	0.751	0.793	0.941	0.105
PCR	0.973	0.455	0.381	0.894	0.935	0.730	0.055
PTR	0.931	0.220	0.253	0.927	0.758	0.402	0.046
Sagittal stratum	0.933	0.392	0.119	0.861	0.823	0.646	0.045
External capsule	0.624	0.485	0.526	0.769	0.669	0.744	0.092
SLF	0.943	0.467	0.438	0.926	0.873	0.816	0.018
SFOF	0.794	0.757	0.774	0.942	0.889	0.906	0.129
UF	0.559	0.804	0.607	0.850	0.349	0.849	0.504
Gray matter							
Whole GM	0.458	0.420	0.325	0.493	0.692	0.173	0.742
Subcortical	0.469	0.365	0.416	0.305	0.782	0.348	0.412
Cortical frontal	0.079	0.403	0.401	0.242	0.849	0.102	0.092
Cortical temporal	0.013	0.095	0.133	0.084	0.395	0.580	0.399
Cortical parietal	0.194	0.469	0.320	0.588	0.428	0.135	0.903
Cortical occipital	0.528	0.482	0.378	0.548	0.753	0.043	0.848
Cortical cingulate	0.295	0.488	0.339	0.596	0.653	0.301	0.574

*ACR*, anterior corona radiata; *ALIC*, anterior limb of the internal capsule; *CC*, corpus callosum; *CST*, corticospinal tract; *GM*, gray matter; *PCR*, posterior corona radiata; *PLIC*, posterior limb of the internal capsule; *PTR*, posterior thalamic radiation; *SCR*, superior corona radiata; *SFOF*, superior fronto-occipital fasciculus; *SLF*, superior longitudinal fasciculus; *UF*, uncinate fasciculus; *WM*, white matter

## Discussion

This study explored the scan–rescan and inter-vendor reproducibility of NODDI metrics (ICVF, ODI, and ISO) obtained using two MR scanners from different vendors in a single-institution setting. Using CoV and ICC analyses, NODDI metrics (ICVF and ODI) in the WM and GM demonstrated comparable scan–rescan and inter-vendor reproducibility with DTI metrics (FA, MD, AD, and RD). In general, however, the reproducibility of ISO was lower compared with the other measured metrics. Also, the inter-vendor reproducibility of all metrics was lower compared with scan–rescan reproducibility.

In contrast to a study by Chung et al. [6], who demonstrated higher scan–rescan CoVs for NODDI metrics than for DTI metrics in the human brain, our study showed that the scan–rescan reproducibility of NODDI metrics using both scanners is comparable with that of DTI metrics (NODDI: ICVF = 0.3–1.5%, ODI = 0.2–3.8% and DTI: FA = 0.2–2.0%, MD 0.2–3.3%, AD = 0.1–2.5%, and RD = 0.2–4.1%). Overall, our study also found lower CoVs of NODDI than those reported by Chung et al. [6] (0.6–7.3%). These results possibly demonstrate that higher-angular resolution pulse sequence (64 directions in this study vs. 20 directions in the previous study) provided more robust diffusion estimates [30]. Our results are consistent with those reported in recent studies that assessed the test–retest reproducibility of DTI metrics acquired for human subjects with 3-T MR scanners using 30 [2] and 64 gradient directions [31] and showed CoVs of < 7% and < 5%, respectively, in whole WM.

DTI and NODDI metrics had higher scan–rescan reproducibility than inter-vendor reproducibility, possibly reflecting cross-scanner differences in the absolute measures of diffusivity, but these results might also be secondary to biological variability [32]. Scan–rescan was done on the same day, but scans on different MR scanners were done on different days. This might have also contributed to the higher inter-vendor differences. Further, the scanners used different head coils (scanner A: 32 channels and scanner B: 64 channels). The differences between the coils affect unfolding methods and performance in parallel and multiband imaging [33]. A larger number of channels in a coil intrinsically lead to higher SNR, particularly in surface regions [34]. In addition, there may have been differences in imaging conditions and environments depending on settings for each vendor that may also explain the inter-vendor differences in terms of SNR. In fact, the SNRs of scanner B for the genu and splenium of the corpus callosum were significantly higher than those of scanner A. Lower SNR has been shown to cause bias in the measurement of diffusion measures [35]. Thus, lower SNR of scanner A may also have contributed to lower inter-vendor reproducibility. Indeed, the scan–rescan CoVs of scanner A were relatively higher than those of scanner B.

Generally, in agreement with the study by Chung et al. [6] investigating scan–rescan reproducibility of NODDI in 1.5 T and 3 T, the reproducibility of NODDI metrics was lower in WM and higher in GM than that of DTI metrics. It has previously been speculated that NODDI metrics are nosier than DTI metrics for modeling WM because NODDI is a more complex model requiring higher b-values [6]. In addition, cardiac pulsation leading to intra-voxel dephasing and inaccurate estimation of anisotropy parameters and tensor orientation possibly increased the variability of NODDI metrics, particularly ODI, in WM [6]. In contrast, NODDI seems to be more robust compared with DTI in the evaluation of GM than on WM, which may be because NODDI metrics serve as a more direct marker for complex and heterogeneous neurobiological features of GM [9, 18].

In line with previous studies [6, 21], ISO in the WM and GM was found to have low scan–rescan and inter-vendor reproducibility compared with the other measured metrics that may be because ISO is highly susceptible to noise [6, 21]. The improvements in SNR are predicted to increase the reproducibility of ISO. In this study, indeed, the reproducibilities of ISO in scanner B are higher than those of scanner A.

Consistent with the observations reported by Zhang et al. [9] and Chung et al. [6], the NODDI maps in our study reflected a spatial pattern of tissue distribution (Figs. 3 and 4), consistent with the known brain anatomy. ICVF, the index of neurite density, values were shown to be lower for GM than for WM and, as expected, ODI, the index of orientation dispersion, were lower in WM but higher in GM (e.g., in WM, the highest ICVF value and lowest ODI value were found in the corpus callosum). Furthermore, FA has been shown to be highly influenced by orientation dispersion [36]. In our study, in line with previous studies [6, 9], ODI and FA exhibited regional variations that are inversely correlated with each other (Figs. 3 and 4).

A major limitation of the present study is the small number of participants who were scanned at a single institution using MRI scanners from only two vendors. To reduce the acquisition time in a clinically feasible manner, DWI data were obtained using a multiband EPI sequence. However, at our institution, the multiband EPI sequence is installed only in MRI scanners from vendors A and B; therefore, we could not expand the study to include additional vendors. Thus, a multi-site study with a larger sample size and more scanners might be needed to demonstrate the robustness of NODDI. In addition, this study was performed using b-values of 1000 and 2000 s/mm^2^, whereas the optimized NODDI protocols use b-values of 711 and 2855 s/mm^2^ (acquired in approximately 30 min). However, Zhang et al. [9] demonstrated that there is no significant loss in the accuracy of the metrics using the current protocol. Furthermore, our protocol can be performed in a shorter time (< 15 min), which makes it more feasible in the clinical setting.

## Conclusion

In this study, NODDI demonstrated excellent scan–rescan reproducibility that was comparable with DTI. However, lower inter-vendor reproducibility of DTI and NODDI in some areas of the brain indicates that data acquired from different MRI scanners should be carefully interpreted.
